# Renin inhibition improves metabolic syndrome, and reduces angiotensin II levels and oxidative stress in visceral fat tissues in fructose-fed rats

**DOI:** 10.1371/journal.pone.0180712

**Published:** 2017-07-10

**Authors:** Chu-Lin Chou, Heng Lin, Jin-Shuen Chen, Te-Chao Fang

**Affiliations:** 1 Division of Nephrology, Department of Internal Medicine, Tri-Service General Hospital, National Defense Medical Center, Taipei, Taiwan; 2 Division of Nephrology, Department of Internal Medicine, Taipei Medical University Hospital, Taipei Medical University, Taipei, Taiwan; 3 Division of Nephrology, Department of Internal Medicine, School of Medicine, College of Medicine, Taipei Medical University, Taipei, Taiwan; 4 Department of Physiology, School of Medicine, College of Medicine, Taipei Medical University, Taipei, Taiwan; 5 Division of Nephrology, Department of Internal Medicine, Wan Fang Hospital, Taipei Medical University, Taipei, Taiwan; Max Delbruck Centrum fur Molekulare Medizin Berlin Buch, GERMANY

## Abstract

Renin–angiotensin system in visceral fat plays a crucial role in the pathogenesis of metabolic syndrome in fructose-fed rats. However, the effects of renin inhibition on visceral adiposity in metabolic syndrome are not fully investigated. We investigated the effects of renin inhibition on visceral adiposity in fructose-fed rats. Male Wistar–Kyoto rats were divided into 4 groups for 8-week experiments: Group Con (standard chow diet), Group Fru (high-fructose diet; 60% fructose), Group FruA (high-fructose diet and concurrent aliskiren treatment; 100 mg/kg body weight [BW] per day), and Group FruB (high-fructose diet and subsequent, i.e. 4 weeks after initiating high-fructose feeding, aliskiren treatment; 100 mg/kg BW per day). The high-fructose diet induced metabolic syndrome, increased visceral fat weights and adipocyte sizes, and augmented angiotensin II (Ang II), NADPH oxidase (NOX) isoforms expressions, oxidative stress, and dysregulated production of adipocytokines from visceral adipose tissues. Concurrent and subsequent aliskiren administration ameliorated metabolic syndrome, dysregulated adipocytokines, and visceral adiposity in high fructose-fed hypertensive rats, and was associated with reducing Ang II levels, NOX isoforms expressions and oxidative stress in visceral fat tissues. Therefore, this study demonstrates renin inhibition could improve metabolic syndrome, and reduce Ang II levels and oxidative stress in visceral fat tissue in fructose-fed rats, and suggests that visceral adipose Ang II plays a crucial role in the pathogenesis of metabolic syndrome in fructose-fed rats.

## Introduction

The prevalence of metabolic syndrome has increased worldwide, and this increase has been linked to the increased intake of high-fructose corn syrup [1]. Metabolic syndrome, a cluster of conditions including increased blood pressure, elevated blood sugar levels, excess body fat around the waistline, and abnormal cholesterol status, which increases a risk of cardiovascular disease, stroke and diabetes [2]. A high-fructose diet (60% fructose) in rodents has also been reported to cause metabolic disturbances, including elevated blood pressure, glucose intolerance, and hyperlipidemia, as well as a dysregulation of the renin–angiotensin system (RAS) [3, 4].

Animal models of fructose-fed rodents have been commonly applied for investigating hypertension and metabolic disturbances [4, 5]. Our recent studies have also observed these phenomena in fructose-fed hypertensive rodents [6, 7]. Nevertheless, although our studies have demonstrated that insulin resistance plays a role in mediating metabolic syndrome, the pathogenesis of these metabolic disturbances has yet to be explicated. Notably, recent studies have demonstrated enhanced nicotinamide adenine dinucleotide phosphate (NADPH) oxidase-mediated oxidative stress and more lipid peroxidation in fructose-fed rats, and lowering NADPH oxidase (NOX)-mediated oxidative stress could ameliorate these metabolic disturbances [8, 9], suggesting that these metabolic disturbances are likely induced by an oxidative stress-mediated process.

Aliskiren, a novel direct renin inhibitor, has been clinically shown to reduce blood pressure in spontaneously hypertensive rats [10], patients with essential hypertension [11], and patients with type 2 diabetes [12]. Our previous studies have shown that aliskiren not only reversed hypertension and endothelial dysfunction but also improved hyperglycemia and dyslipidemia in fructose-fed hypertensive rats [6, 7]. In addition, our recent study reported that calcitriol reduced the visceral fat pad weight and adipocyte size by reducing adipose angiotensin II (Ang II) levels in fructose-fed hypertensive rats [13]. Recently, a study reported that Ang II could cause NOX-dependent increases in adipose oxidative stress and inflammation in transgenic mice overexpressing angiotensinogen [14]. Moreover, Farina et al. showed that apocynin, a well-known inhibitor of NOX, could improve adipose leptin expression, fatty acid composition, fat pad weight, and size of adipocytes derived from visceral adipose tissues in rats receiving 10% (w/v) fructose in their drinking water for 3 weeks [15]. Currently, the beneficial effects of apocynin on oxidative stress in visceral adipose tissue have not been examined in patients with metabolic syndrome, because the use of apocynin in clinical practice has not been approved. Therefore, studies should focus on examining other potential drugs that have been extensively used in clinical practice and can reduce Ang II concentration or block its signaling, subsequently reducing NOX activity and oxidative stress. A direct renin inhibitor, aliskiren, has been extensively applied in rats with spontaneous hypertension [10] and can block Ang II production [16]. Recently reported, aliskiren have the beneficial effects on improving insulin sensitivity, hepatic steatosis, peripheral fat mass, and oxidative stress markers in rodents with metabolic syndrome [17, 18]. Further, the effects of renin inhibition on visceral adiposity in metabolic syndrome are currently under investigation. Therefore, we examined the effects of the direct renin inhibitor aliskiren on Ang II, oxidative stress signaling, and adipocytokines in visceral adipose tissue in fructose-fed hypertensive rats.

## Materials and methods

### Animals

All experimental procedures were approved by the Institutional Animal Care and Use Committee of Taipei Medical University (Protocol Number: LAC-2015-0041) and in strict accordance with the recommendations in the Guide for the Care and Use of Laboratory Animals of the National Institutes of Health. Male Wistar–Kyoto rats with the initial weight of 200–230 g, were conducted for the experiments. The rats were kept in individual cages in a room with air maintained at 24–27°C, humidity of 50%–80%, and a 12 h light/dark cycle.

The high-fructose diet (Harlan Teklad, Madison, WI, USA) was constituted of 60% fructose, 21% protein, 5% fat, 8% cellulose, and a standard vitamin and mineral mixture. The standard chow diet was composed of 50% starch, 21% protein, 4% fat, 4.5% cellulose, and a standard vitamin and mineral mixture.

### Experimental protocols

After an adaptation period of 1 week, the male Wistar–Kyoto rats derived from the National Laboratory Animal Center in Taiwan were divided into the following 4 groups (n = 6 for each group) for the 8-week experiments: Group Con, comprising control rats that were fed the standard chow diet for 8 week; Group Fru, comprising rats that were fed the high-fructose diet for 8 week; Group FruA, comprising rats that were fed the high-fructose diet and were coinfused with aliskiren (100 mg/kg body weight [BW] per day; a kind gift from Novartis Pharmaceuticals, Basel, Switzerland) via a subcutaneous osmotic minipump for 8 week; and Group FruB, comprising rats that received the same treatment as Group Fru, but aliskiren (100 mg/kg BW per day) was administered 4 weeks after the initiation of high-fructose feeding. The dose of aliskiren used in the current study is based on other and our previous studies [6, 7, 10], which have revealed that aliskiren treatment at 100 mg/kg per day significantly lowered blood pressure in hypertensive rats. The first day of fructose feeding was defined as Day 1. BW was measured twice a week, and systolic blood pressure (SBP) was also measured twice a week through the tail-cuff method. To measure the levels of triglycerides, total cholesterol, glucose, insulin, creatinine, and adipocytokines (adiponectin, leptin, resistin, and visfatin), blood samples (1 mL) were collected after 12 h of fasting on Day 0, at the midpoint of the experimental period (Day 28) from the femoral artery, and at the end of the study (Day 56) from the heart under adequate anesthesia. The rats were anesthetized by intraperitoneal injection with ketamine (60 mg/kg BW) and xylazine (7.5 mg/kg BW) on Day 0 and Day 28 and sodium pentobarbital (40 mg/kg BW) on Day 56. On Day 56, the rats were sacrificed by exsanguination, and blood was collected from the heart; their tissues were rapidly collected under deep anesthesia. Blood were collected in the EDTA-containing tubes and immediately placed in an ice bath (2–4°C).

### SBP measurements

The rats were allowed ad libitum access to water and were kept in a quiet area before SBP measurement at 9 AM. We used the tail-cuff method without preheating to reliably measure SBP with a programmed electro-sphygmomanometer (MK-2000ST, Muromachi, Tokyo, Japan), as described in our previous [6, 7, 19, 20] and other studies [21, 22]. The mean of 6 consecutive readings was determined as the SBP value for each rat for that day, and SBP was checked twice a week during the adaptation (1 week) and experimental (8 weeks) periods.

### Osmotic minipump installation

Osmotic minipump installation was conducted according to our previous studies [6, 7, 13]. An osmotic minipump (No. 2002, 14 days of active life, Alza Corp) was filled with aliskiren dissolved in deionized water (Novartis Pharmaceuticals Co., NJ, USA), which was implanted subcutaneously in the rats under brief anesthesia with sodium pentobarbital (40 mg/kg BW, intraperitoneal injection).

#### Laboratory measurements

Blood samples were immediately centrifuged at 4000 *g* for 10 min at 4°C. These samples were aliquoted and applied for immediate assays of glucose, insulin, triglycerides, total cholesterol, creatinine, and adipocytokines. Serum glucose was measured using an Accu-Check Advantage Blood Glucose Monitor (Roche Diagnostic Corporation, Indianapolis, IN, USA). Serum insulin was measured using a Mercodia Ultrasensitive Rat Insulin ELISA (Mercodia AB, Uppsala, Sweden). Serum triglycerides, total cholesterol, and creatinine were determined through standard methods by using a COBAS Integra 800 analyzer (Roche Diagnostics, Indianapolis, IN). Serum adipocytokines, such as adiponectin (ALPCO Diagnostics, Salem, NH, USA), leptin (R&D Systems, Minneapolis, MN, USA), visfatin (Cayman Chemical, Ann Arbor, MI, USA), and resistin (BioVendor, Brno, Czech Republic), were measured using commercially available enzyme-linked immunosorbent assay kits.

### Measurement of visceral fat pad weights and adipocyte sizes

On Day 56, retroperitoneal, mesenteric, and epididymal fat pads were rapidly dissected, weighed, and stored at −80°C. We measured the weights of the fat pads, which were normalized to BW (gram/gram, %), and 3-mm-thick paraffin-embedded specimens of the fat pads were stained with hematoxylin–eosin. We measured adipocyte sizes, which were counted under a microscope. The average of 50 measurements of the adipocyte size (diameter, μm) was measured as an individual value, according to the method of Furuhashi et al. [23].

### Analysis of adipose Ang II levels

Tissue samples (0.3 mg) derived from the retroperitoneal, mesenteric, and epididymal fat pads (white adipose tissue), the periaortic fat pad from thoracic aorta (brown adipose tissue), and the kidney were homogenized at 4°C in tissue extraction buffer (0.6 mL) for 15 minutes to determine the levels of Ang II, as reported by our previous study in the extraction process [13]. Subsequently, for determining adipose Ang II levels, 0.5 mL of the supernatant below the fat layer was assayed by applying a quantitative sandwich enzyme immunoassay technique executed through a commercially available Ang II kit (SPI Bio, Montigny Le Bretonneux, France). Tissue protein quantitation was performed using a Pierce™ BCA Protein Assay Kit (Thermo Scientific, Waltham, MA). Adipose Ang II values are expressed as picogram per milligram of protein.

### Analysis of oxidative stress in visceral adipose tissues

To measure the levels of the isoforms of NOX family in retroperitoneal, mesenteric, and epididymal fat, these adipose tissues were lysed, and proteins were separated through SDS-PAGE and transferred onto a nitrocellulose membrane. The membranes were incubated overnight at 4°C with the following primary antibodies: anti-NOX1 (Abcam, Cambridge, UK), anti-NOX2 (Abcam, Cambridge, UK), anti-NOX4 (Abcam, Cambridge, UK). The washed membranes were incubated with horseradish peroxidase-conjugated secondary antibody. Subsequently, the membranes were incubated with horseradish peroxidase-conjugated secondary antibodies for 1 h at room temperature. The membranes were re-probed for beta-actin to verify the uniformity of protein loading. Bands were visualized using autoradiography and quantified using commercially available software. The results were normalized to the optical density of a standard sample. The independent samples were analyzed at least six times for each experimental condition.

Adipose superoxide dismutase (SOD) activity (Cayman Chemical) was determined according to the procedure of Galinier et al. [24]. The lipid peroxide level, a marker of oxidative stress, in the visceral adipose tissue was assessed by measuring thiobarbituric acid reactive substances (TBARS) through a commercially available kit (Cell Biolabs, San Diego, CA), according to the method of Rebolledo and Yagi [25, 26]. TBARS values are expressed as nanomoles per milligram of protein.

### Analysis of adipocytokines in visceral adipose tissues

To measure the levels of adipocytokines in retroperitoneal, mesenteric, and epididymal fat, these adipose tissues were lysed, and proteins were determined through western blotting, as described above in the analysis of oxidative stress in visceral adipose tissues. The membranes were incubated overnight at 4°C with the following primary antibodies: anti-adiponectin (Genetex, Irvine, CA, USA), anti-leptin (Santa Cruz Biotechnology, Santa Cruz, CA, USA), anti-visfatin (Genetex, Irvine, CA), and anti-resistin (Santa Cruz Biotechnology, Santa Cruz, CA). The results were normalized to the optical density of a standard sample. The independent samples were analyzed at least six times for each experimental condition.

### Statistical analyses

All results were expressed as means ± standard deviation. One-way ANOVA followed by Newman-Keuls’s post hoc test or one-way repeated-measures ANOVA was performed to compare all groups. The changes in body weight and food intake were analyzed by repeated measures ANOVA. Two-way ANOVA (the first factor being the treatment group and the second the time period) considering type of diet and drug treatment as factors was performed for comparison between groups, such as analyzing SBP. We also performed the Student t test for unpaired data when appropriate. A P value less than 0.05 was considered statistically significant.

## Results

### Effects of aliskiren on SBP in fructose-fed rats

**Fig 1:**
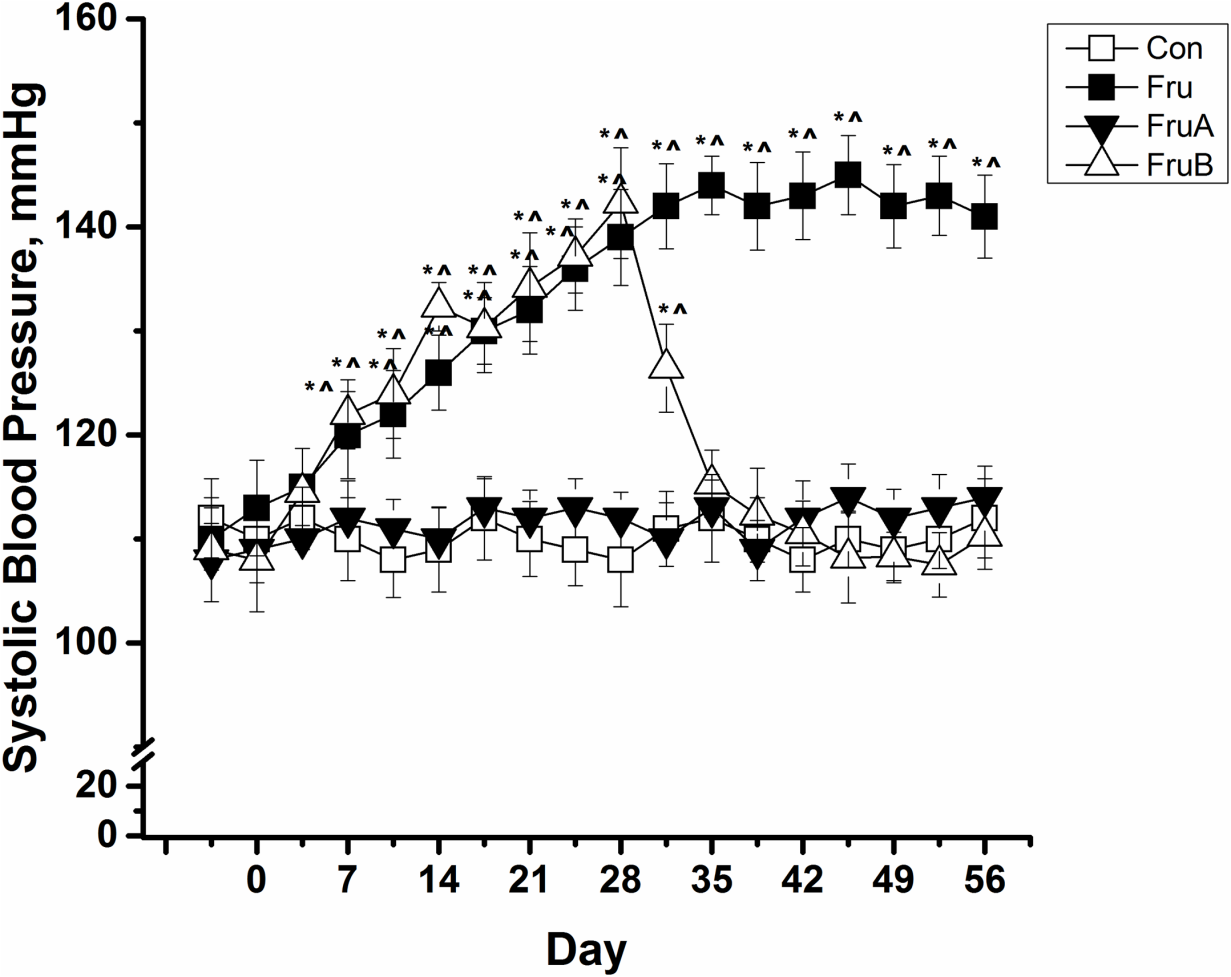
Changes in systolic blood pressure in control and fructose-fed rats with or without aliskiren treatment. Con: control rats were fed the normal chow diet; Fru: rats were fed the high-fructose diet for 8 weeks; FruA: rats received the same treatment as Group Fru, and aliskiren was concurrently administered; FruB: rats received the same treatment as Group Fru, and aliskiren was administered 4 weeks after the initiation of high-fructose feeding. Values are expressed as means ± standard deviation (SD) mean of six independent samples. * and ^ denote *P* < 0.05 versus a pre-fructose period and control rats at the corresponding time point, respectively. N = 6 for each group.

Fig 1 presents SBP changes in control and fructose-fed rats with or without aliskiren treatment. The high-fructose diet significantly induced the rise of SBP levels of rats from the baseline value of 110 ± 3 mm Hg to 139 ± 5 and 141 ± 4 mm Hg in the rats on Day 28 and Day 56, respectively. Furthermore, concurrent 8-week aliskiren treatment prevented the increase in SBP in the fructose-fed rats. Subsequent 4-week aliskiren treatment also ameliorated the SPB levels in the fructose-fed hypertensive rats, which exhibited SBP levels similar to those exhibited by the control rats.

### Effects of aliskiren on glucose, insulin, triglycerides, cholesterol and adipocytokines concentrations in serum of fructose-fed rats

Table 1 presents a summary of the effects of a high-fructose diet alone and in combination with aliskiren treatment on serum glucose, insulin, triglycerides, total cholesterol, creatinine, and adipocytokines. Throughout the study period, high-fructose feeding significantly increased serum glucose, insulin, triglyceride, and total cholesterol levels, compared with those in the control rats. Concurrent 8-week aliskiren treatment improved the rise of serum glucose, insulin, triglycerides, and total cholesterol levels in the fructose-fed rats. Moreover, subsequent 4-week aliskiren treatment demonstrated similar effects in the fructose-fed rats. Serum creatinine, which represents kidney function, did not change among these 4 groups throughout the study period.

**Table 1:** Effects of a high-fructose diet alone and in combination with aliskiren treatment on serum glucose, insulin, triglycerides, total cholesterol, creatinine, and adipocytokines.

*Group*	*Con*	*Fru*	*FruA*	*FruB*
Glucose, mmol/L				
Day 0	6.45 ± 0.70	6.36 ± 0.75	6.32 ± 0.56	6.38 ± 0.54
Day 28	6.40 ± 0.41	10.23 ± 0.79^*^^^^	6.42 ± 0.35^#^	10.56 ± 1.10^*^^^^
Day 56	6.62 ± 0.52	10.73 ± 0.39^*^^^^	6.92 ± 0.65^#^	7.04 ± 0.58^#^
Insulin, pmol/L				
Day 0	159.3 ± 21.5	151.1 ± 25.1	152.6 ± 24.8	155.2 ± 23.3
Day 28	157.8 ± 18.2	293.0 ± 30.8^*^^^^	158.6 ± 26.9^#^	289.6 ± 28.2^*^^^^
Day 56	151.2 ± 25.4	297.8 ± 21.3^*^^^^	168.2 ± 22.8^#^	181.2 ± 25.2^#^
Triglycerides, mmol/L				
Day 0	1.56 ± 0.09	1.58 ± 0.12	1.58 ± 0.10	1.52 ± 0.08
Day 28	1.60 ± 0.10	3.11 ± 0.21^*^^^^	1.62 ± 0.12^#^	3.12 ± 0.19^*^^^^
Day 56	1.55 ± 0.08	3.29 ± 0.21^*^^^^	1.68 ± 0.20^#^	1.72 ± 0.13^#^
Total cholesterol, mmol/L				
Day 0	2.25 ± 0.21	2.28 ± 0.25	2.23 ± 0.24	2.20 ± 0.30
Day 28	2.28 ± 0.42	3.21 ± 0.28^*^^^^	2.36 ± 0.26^#^	3.23 ± 0.21^*^^^^
Day 56	2.30 ± 0.25	3.26 ± 0.25^*^^^^	2.51 ± 0.22^#^	2.68 ± 0.25^#^
Creatinine, μmol/L				
Day 0	41.55 ± 6.40	39.63 ± 5.40	40.42 ± 5.93	40.26 ± 5.64
Day 28	40.31 ± 4.51	41.52 ± 4.65	43.62 ± 6.03	41.87 ± 5.52
Day 56	41.62 ± 6.24	42.23 ± 5.63	43.82 ± 5.21	42.74 ± 5.04
Adiponectin, ng/mL				
Day 0	12 673 ± 571	12 516 ± 997	12 022 ± 516	12 496 ± 985
Day 28	11 812 ± 919	7488 ± 812^*^^^^	11 088 ± 678^#^	7698 ± 699^*^^^^
Day 56	12 527 ± 650	7158 ± 990^*^^^^	11 030 ± 976^#^	9618 ± 727^*^^^^^#^
Leptin, ng/mL				
Day 0	295 ± 71	294 ± 23	232 ± 37	250 ± 46
Day 28	634 ± 218^*^	1368 ± 227^*^^^^	531 ± 139^*^^#^	1321 ± 220^*^^^^
Day 56	507 ± 230^*^	1347 ± 223^*^^^^	440 ± 198^*^^#^	648 ± 216^*^^#^
Resistin, ng/mL				
Day 0	22.49 ± 2.01	20.98 ± 1.97	22.37 ± 3.41	23.43 ± 1.41
Day 28	22.54 ± 1.32	31.83 ± 3.15^*^^^^	21.82 ±3.33^#^	30.32 ± 3.44^*^^^^
Day 56	20.87 ± 3.56	30.48 ± 3.73^*^^^^	23.48 ±1.92^#^	22.72 ± 2.62^#^
Visfatin, ng/mL				
Day 0	11.54 ± 1.15	12.36 ± 2.78	11.56 ± 2.14	10.98 ± 2.37
Day 28	10.28 ± 2.42	22.42 ± 3.27^*^^^^	10.81 ±2.51^#^	21.33 ± 2.72^*^^^^
Day 56	10.98 ± 2.51	21.67 ± 2.94^*^^^^	11.32 ± 2.75^#^	13.67 ± 2.75^#^

Con: control rats were fed a normal chow diet; Fru: rats were fed a high-fructose diet for 8 weeks; FruA: rats received the same treatment as Group Fru, and aliskiren was concurrently administered; FruB: rats received the same treatment as Group Fru, and aliskiren was administered 4 weeks after the initiation of high-fructose feeding. Values are expressed as means ± SD.

* denotes *P* < 0.05 versus pre-fructose period.

^ and # denote *P* < 0.05 versus control rats and fructose-fed rats at the corresponding time point, respectively. N = 6 for each group.

Throughout the study period, high-fructose feeding significantly reduced serum adiponectin levels and increased serum leptin, resistin, and visfatin levels, compared with those in the control rats. Concurrent 8-week aliskiren treatment ameliorated the lower adiponectin levels and the higher leptin, resistin, and visfatin levels in the fructose-fed rats relative to the baseline levels observed in the rats fed the standard chow diet. Subsequent 4-week aliskiren treatment also demonstrated similar effects in the fructose-fed rats.

### Effects of aliskiren on visceral fat pad weights, adipocyte sizes, and adipose Ang II levels in fructose-fed rats

**Fig 2:**
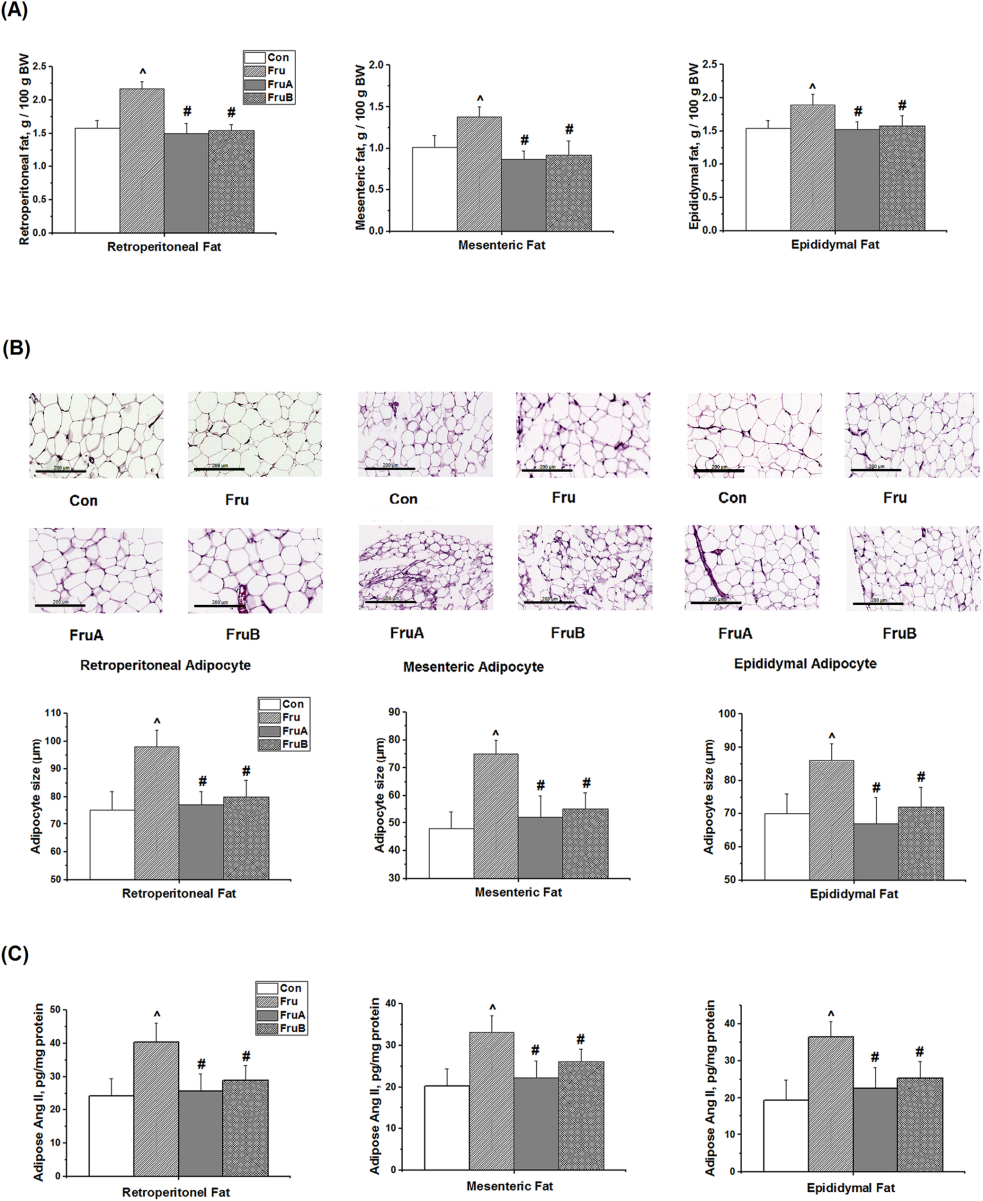
**Effects of aliskiren on (A) the ratio of visceral fat pad weight over the whole body weight (BW) (gram/gram, %), (B) the histological photographs and sizes of adipocyte tissues, and (C) adipose angiotensin II levels from retroperitoneal, mesenteric, and epididymal fat in fructose-fed hypertensive rats.** Con: control rats were fed the normal chow diet; Fru: rats were fed the high-fructose diet for 8 weeks; FruA: rats received the same treatment as Group Fru, and aliskiren was concurrently administered; FruB: rats received the same treatment as Group Fru, and aliskiren was administered 4 weeks after the initiation of high-fructose feeding. Scale bar: 200 μm. Values are expressed as means ± SD mean of six independent samples. ^ and # denote *P* < 0.05 versus control rats and fructose-fed rats, respectively. N = 6 for each group.

No significant differences were observed in food intake and BW gain among the 4 groups at the corresponding time points throughout the study period (S1 Fig). Compared with the control rats, the weight ratio of the retroperitoneal, mesenteric, and epididymal fat pad weights over the whole BW (gram/gram, %) was higher in the fructose-fed rats (2.17% ± 0.11% vs. 1.58% ± 0.12%, *P* < 0.05; 1.38% ± 0.12% vs. 1.01% ± 0.15%, *P* < 0.05; and 1.89% ± 0.16% vs. 1.54% ± 0.12%, *P* < 0.05, respectively; Fig 2A). Further, compared with fructose-fed rats without aliskiren treatment, concurrent and subsequent aliskiren treatments significantly reduced the weight ratio of the retroperitoneal, mesenteric, and epididymal fat pad weights over the whole BW (gram/gram, %).

The mean sizes of adipocytes derived from the visceral fat pads were noticeably larger in the fructose-fed rats than in the control rats (Fig 2B). The sizes of the adipocytes derived from the retroperitoneal, mesenteric, and epididymal fat pads were significantly larger in the high-fructose fed rats than in the control rats. Concurrent and subsequent aliskiren treatments significantly reduced the sizes of adipocytes derived from the retroperitoneal, mesenteric, and epididymal fat pads in the fructose-fed rats.

Fig 2C shows the effect of aliskiren on the levels of Ang II derived from the retroperitoneal, mesenteric, and epididymal fat pads (white adipose tissues) in the fructose-fed hypertensive rats. The Ang II levels of the periaortic brown adipose tissue from the thoracic aorta and the kidney in the control rats are showed as the references in S1 Table. High-fructose feeding increased Ang II levels from the visceral adipose tissues of the rats. Concurrent 8-week aliskiren treatment prevented Ang II levels from the visceral adipose tissues, including retroperitoneal, mesenteric, and epididymal fat, in the fructose-fed rats. Similarly, subsequent 4-week aliskiren treatments reversed the increased Ang II levels from the visceral adipose tissues.

### Effects of aliskiren on oxidative stress in visceral adipose tissues of fructose-fed rats

**Fig 3:**
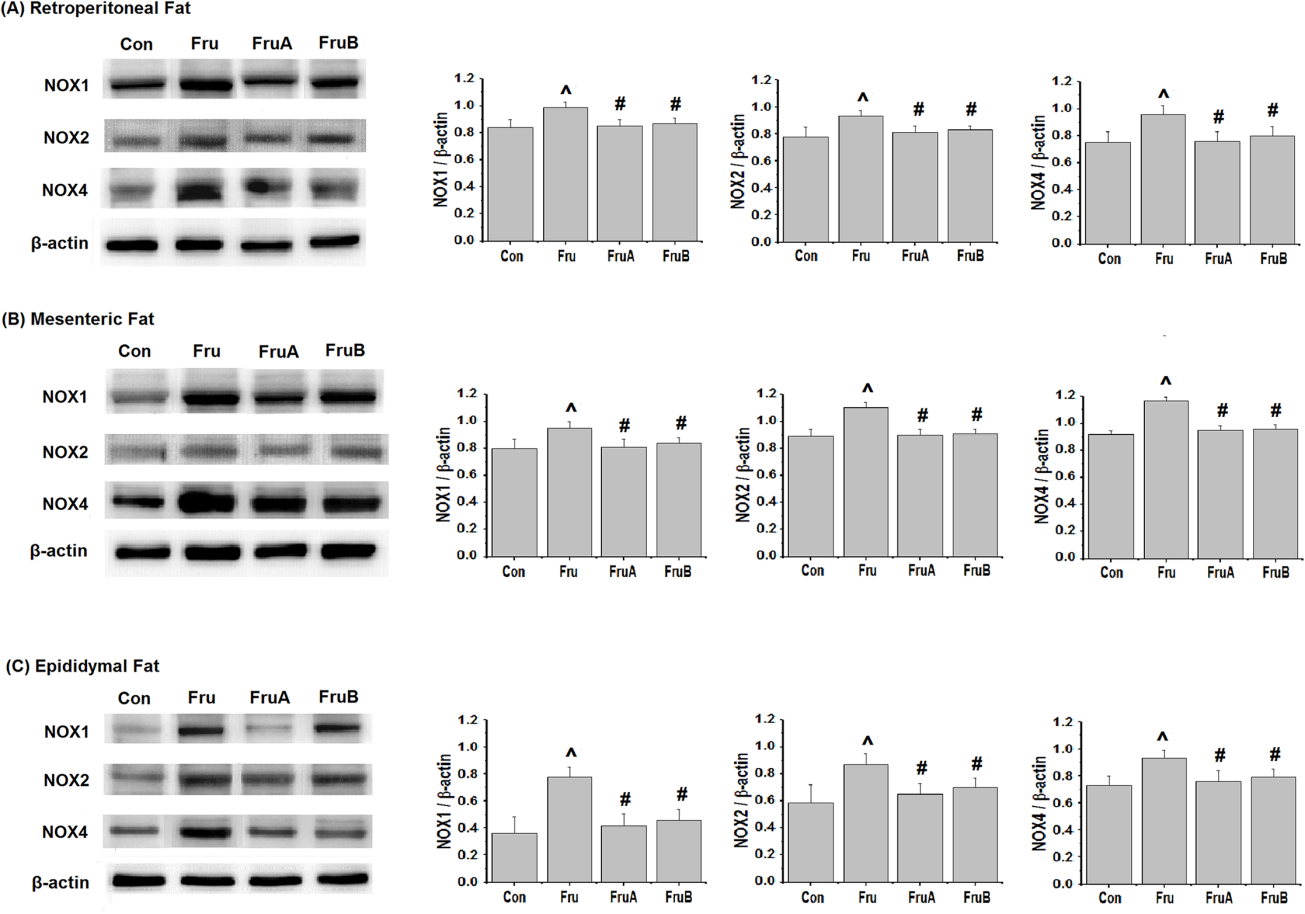
**Effects of aliskiren on visceral adipose expression of the isoforms (A) NOX1, (B) NOX2, and (C) NOX4 of NOX family in fructose-fed hypertensive rats.** Con: control rats were fed the normal chow diet; Fru: rats were fed the high-fructose diet for 8 weeks; FruA: rats received the same treatment as Group Fru, and aliskiren was concurrently administered; FruB: rats received the same treatment as Group Fru, and aliskiren was administered 4 weeks after the initiation of high-fructose feeding. Values are expressed as means ± SD mean of six independent samples. ^ and # denote *P* < 0.05 versus control rats and fructose-fed rats, respectively. N = 6 for each group.

Fig 3 illustrates the effects of aliskiren on levels of NOX isoforms in the visceral adipose tissues of the fructose-fed hypertensive rats. Fructose-feeding significantly increased NOX1, NOX2, and NOX4 expression levels in the retroperitoneal, mesenteric, and epididymal fat pads. However, concurrent 8-week and subsequent 4-week aliskiren treatment reduced the expression levels of NOX1, NOX2 and NOX4.

**Fig 4:**
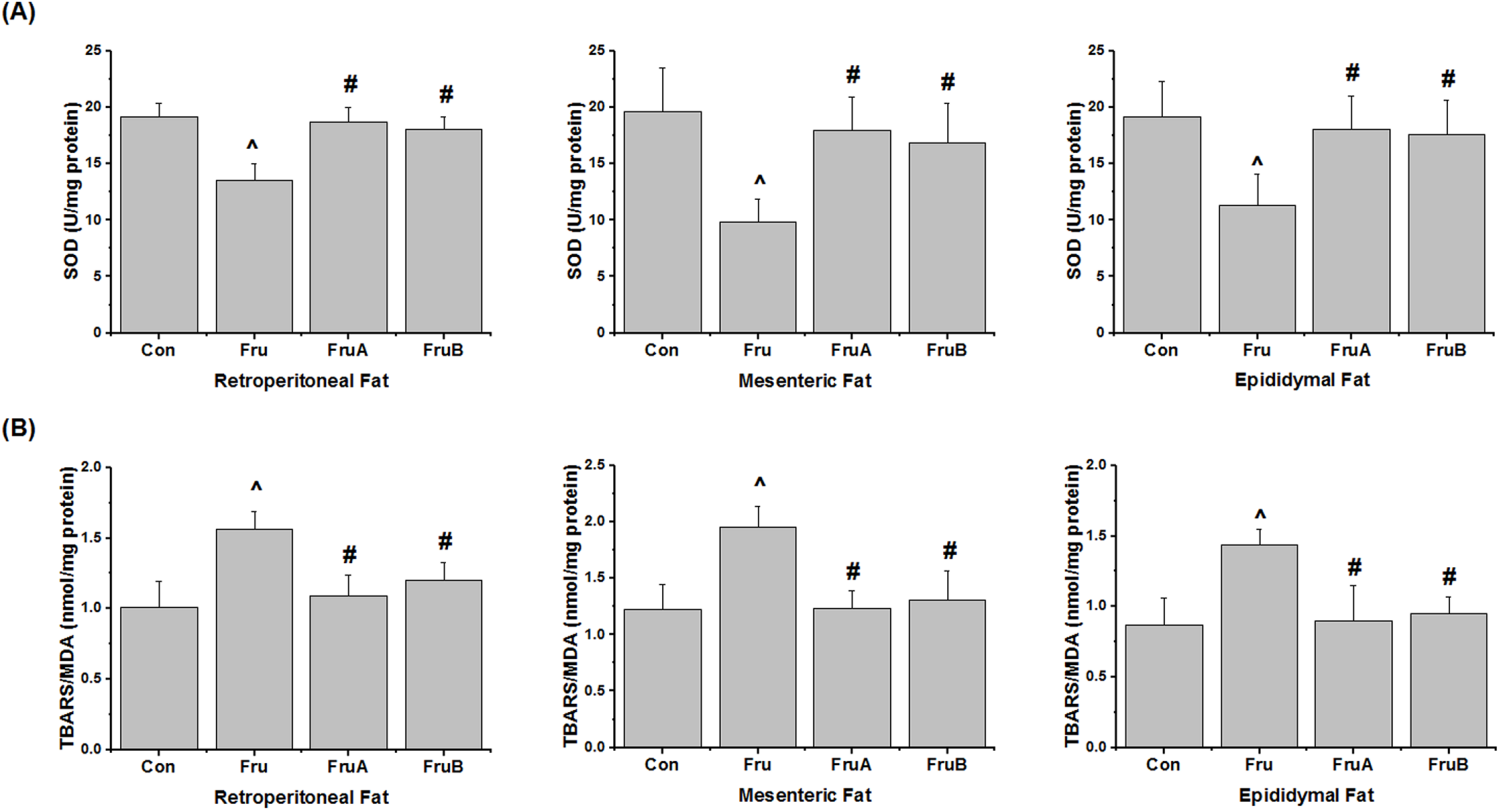
**Effects of aliskiren on markers of oxidative stress in visceral adipose tissues, namely (A) superoxide dismutase (SOD) and (B) thiobarbituric acid reactive substances (TBARS), in fructose-fed hypertensive rats.** Con: control rats were fed the normal chow diet; Fru: rats were fed the high-fructose diet for 8 weeks; FruA: rats received the same treatment as Group Fru, and aliskiren was concurrently administered; FruB: rats received the same treatment as Group Fru, and aliskiren was administered 4 weeks after the initiation of high-fructose feeding. Values are expressed as means ± SD mean of six independent samples. ^ and # denote *P* < 0.05 versus control rats and fructose-fed rats, respectively. N = 6 per group.

Fig 4 presents effects of aliskiren on visceral adipose SOD activity and lipid peroxide levels of TBARS in the fructose-fed rats. The visceral adipose SOD activity were reduced by the high-fructose diet and subsequently increased by concurrent 8-week and subsequent 4-week aliskiren treatments, compared with the activity observed for the high-fructose diet without any aliskiren treatment. Moreover, the rats on the high-fructose diet exhibited higher visceral adipose lipid peroxide levels than did the control rats. Concurrent and subsequent aliskiren treatments reduced the lipid peroxide levels, compared with those examined in rats on the high-fructose diet without aliskiren treatment.

#### Effects of aliskiren on visceral adipose adipocytokines in fructose-fed rats

**Fig 5:**
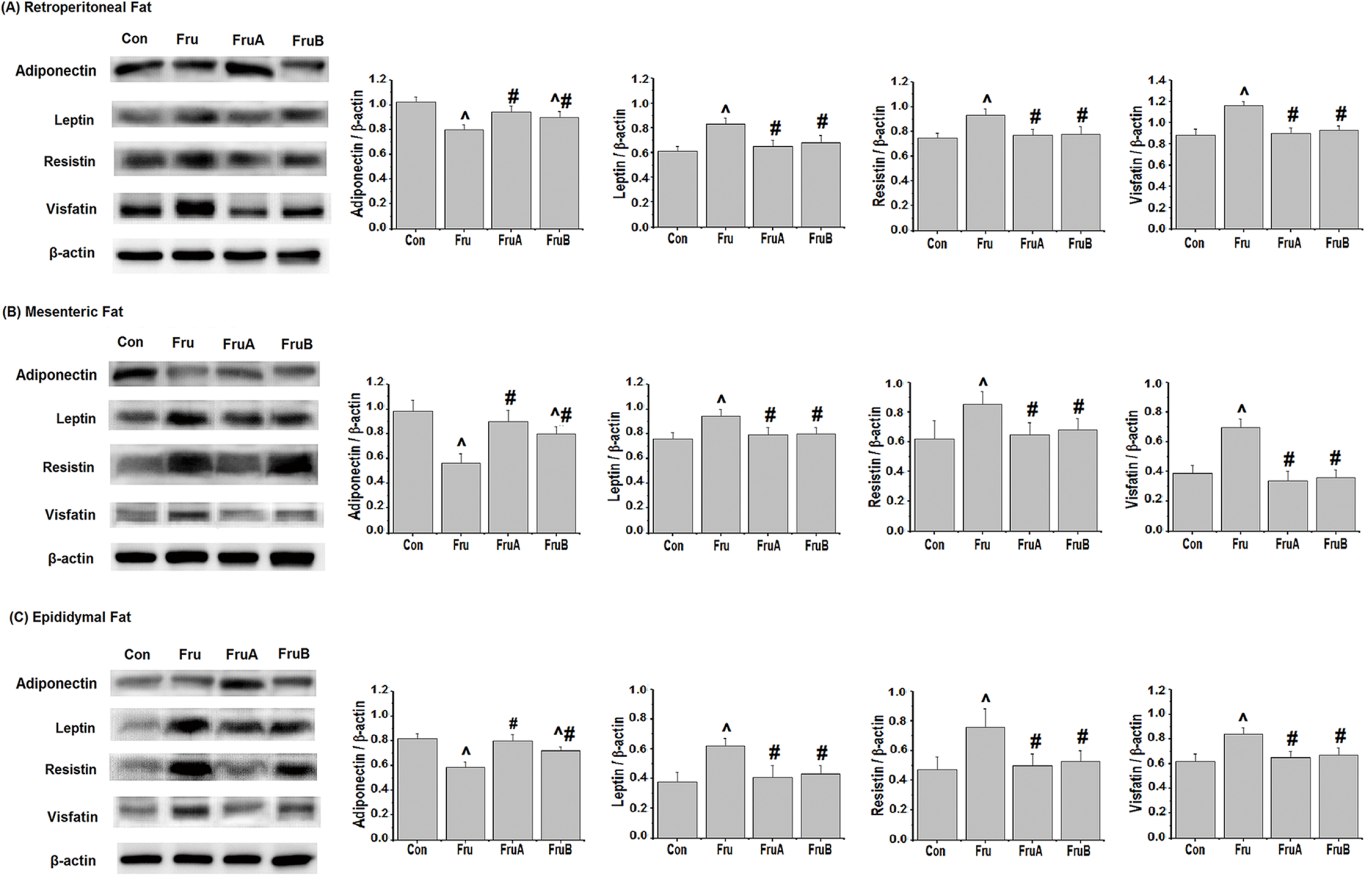
**Effects of aliskiren on the expression of adiponectin, leptin, resistin, and visfatin in (A) retroperitoneal fat, (B) mesenteric fat, and (C) epididymal fat of fructose-fed hypertensive rats.** Con: control rats were fed the normal chow diet; Fru: rats were fed the high-fructose diet for 8 weeks; FruA: rats received the same treatment as Group Fru, and aliskiren was concurrently administered; FruB: rats received the same treatment as Group Fru, and aliskiren was administered 4 weeks after the initiation of high-fructose feeding. Values are expressed as means ± SD mean of six independent samples. ^ and # denote *P* < 0.05 versus control rats and fructose-fed rats, respectively. N = 6 per group.

Fig 5 depicts effects of aliskiren on the expression levels of adiponectin, leptin, resistin, and visfatin in the visceral adipose tissues of the fructose-fed hypertensive rats. The high-fructose diet reduced adiponectin expression and increased the leptin, resistin, and visfatin expression levels in the retroperitoneal, mesenteric, and epididymal fat pads. Concurrent 8-week aliskiren treatment improved the expression levels of these adipocytokines in the visceral adipose tissues of fructose-fed rats. Subsequent 4-week aliskiren treatments also had similar effects.

## Discussion

In this study, our major findings are as follows: First, high-fructose feeding caused systolic hypertension and increased serum glucose, insulin, triglycerides, and total cholesterol levels, consistent with the findings of other [27, 28] and our previous reports [6, 7]. Second, compared with control rats, high-fructose feeding significantly reduced serum and visceral adipose adiponectin levels; increased serum and visceral adipose leptin, resistin, and visfatin levels; increased the visceral fat pad weight and adipocyte size; elevated Ang II and NOX isoforms expressions; and caused oxidative stress (reduced adipose SOD activity and increased adipose lipid peroxide). Third, concurrent and subsequent aliskiren treatments ameliorated SBP, hyperglycemia, and dyslipidemia, as well as increased adipose SOD activity and reduced adipose lipid peroxide and visceral adipose NOX isoforms expressions, and improved dysregulated adipocytokines and adipose hypertrophy. All these data suggest that renin-inhibited pathways have the benefits on improving adipocyte oxidative stress and adiposity with regard to the increased worldwide uptake of high-fructose corn syrup.

Previous studies [29, 30] and our studies [6, 7, 13] have shown that RAS plays a major role in the pathogenesis of blood pressure and vascular dysfunction in rats with high fructose-induced hypertension. High-fructose feeding in rats caused elevated plasma Ang II, increased the mRNA expression level of the aortic tissue Ang II type 1 receptor, caused high blood pressure, impaired endothelial nitric oxide (NO) synthase, and caused vascular dysfunction [29–31]. Furthermore, the RAS blockade of angiotensin-converting enzyme (ACE) inhibitors or the Ang II type 1 receptor blocker (ARB) has been reported to reduce blood pressure and reconstruct the natural process of endothelial NO synthase in fructose-fed rats [31, 32].

In this study, aliskiren improved glucose intolerance and insulin resistance in the fructose-fed rats. Previous studies have shown that a direct renin inhibitor increased pancreatic islet function and upregulated insulin action on skeletal muscle glucose transport, further improving glucose intolerance and insulin resistance through the downregulation of tissue Ang II and Ang II type 1 receptor [33, 34]. Moreover, this study shows that aliskiren ameliorated dyslipidemia, consistent with the findings of other studies demonstrating that aliskiren improved hypercholesterol and hypertriglyceride levels in models of insulin resistance [35, 36]. The effects of ACE inhibitors and ARB on dyslipidemia in the fructose-fed rats observed in this study are similar to those observed in previous studies [23, 37].

Several studies have shown that adipose tissue contributes to the modulation of changes in a variety of bioactive adipocytokines, such as adiponectin, leptin, visfatin, and resistin, leading to vascular injury and target organ damage [38, 39]. In the current study, the high-fructose diet significantly reduced serum adiponectin levels and increased serum leptin, resistin, and visfatin levels by increasing adipose Ang II expression. By contrast, concurrent and subsequent aliskiren treatments increased serum adiponectin levels and reduced the serum levels of harmful adipocytokines, such as leptin, resistin, and visfatin, by reducing adipose Ang II levels in the fructose-fed rats. Several studies have also revealed that Ang II signaling plays a role in the dysregulated production of adipocytokines, and that ARB can improve the dysregulated production of adipocytokines from plasma and tissues in fructose-fed rats [40–42]. For example, Ran et al. showed that Ang II infusion in fructose-fed rats reduced the plasma adiponectin level through the Ang II type 1 receptor, resulting in impaired insulin sensitivity [40]. Moreover, Umeda et al. reported that ARB reduced plasma leptin and leptin mRNA in adipose tissues in rats fed sucrose-rich chow [42].

Regarding the roles of visceral adipose Ang II in the pathogenesis of fructose-induced metabolic syndrome, this study shows that high-fructose feeding increased visceral adipose Ang II expression, NOX isoforms expressions, oxidative stress (reducing SOD activity and increasing lipid peroxide levels), dysregulated adipocytokines, and visceral adiposity. Furthermore, aliskiren treatment ameliorated dysregulated adipocytokine and was associated with reducing angiotensin II levels, NOX isoforms expressions and oxidative stress. Several animal studies have also demonstrated that increasing NOX activity in adipose tissues caused oxidative stress and dysregulated production of adipocytokines [4, 43–45]. Several studies have also reported that ARB could ameliorate oxidative stress in adipose tissues by reducing NOX activity in both insulin-resistant and diet-induced obese mice [14, 46, 47]. For example, Kalupahana et al. demonstrated that the major sources of oxidative stress in adipose tissues are the activation of membrane-associated NADPH-oxidases by Ang II stimulation in transgenic mice overexpressing angiotensinogen in adipose tissues, and they demonstrated that RAS overactivation causes systemic insulin resistance [14]. In the in vitro study of Caminhotto et al., lipolysis, lipogenesis and glucose oxidation capacities of different RAS blockers (aliskiren, captopril, and losartan) were investigated through co-incubated isolated primary adipocytes and different RAS blockers in vitro for 24 h [48]. Concerning the effect of aliskiren treatment, aliskiren treatment only increased glucose oxidation capacities following insulin stimulation in fat cells, which implies that in vitro aliskiren treatment has an improvement of oxidative stress in isolated primary adipocytes. Also, Caminhotto et al. suggested that the in vivo effects of aliskiren treatment on adipose oxidative stress merit to be further clarified [48]. In our in vivo study, the data showed that aliskiren treatment reduced visceral adipose Ang II and NOX isoforms expressions, increased adipose SOD activity, and diminished adipose lipid peroxide in fructose-fed rats, which indicated that in vivo aliskiren treatment had the improvement of oxidative stress in visceral adipocytes. The benefits of aliskiren treatment on oxidative stress in our study also consisted with Rabie et al., which reported that aliskiren treatment ameliorates oxidative stress markers, such as malondialdehyde, nitric oxide, glutathione levels and catalase activity in fructose-fed rats [17].

Ang II is one of the potent stimuli of NADPH oxidase activity. The activation of the NADPH oxidase by Ang II is partly mediated by protein kinase C (PKC), the inhibitor of PKC could be effective to reduce NADPH oxidase activity in conditions where Ang II is increased [49]. In hypertensive rats, which were induced by chronic Ang II infusion, the expression of NOX1 and p22phox mRNA in aortas is elevated [50]. Moreover, the increase in blood pressure caused by Ang II infusion is markedly reduced in p47phox-/- mice [51]. Thus, Guzik et al. proposed that Ang II activates PKC leading to phosphorylation of p47phox allowing it’s binding to p22phox, then causing NADPH oxidase activity[52]. In the future, the determination of NADPH oxidase activation that is phosphorylation of p47phox by PKC in adipose tissues of fructose-fed rats merits to be conducted in the following studies.

Our results reveal that the high-fructose diet increased the weight of visceral fat pads (including retroperitoneal, mesenteric, and epididymal fat) and adipocyte sizes in the rats, and that aliskiren treatment reduced visceral fat pad weights and adipocyte sizes through the downregulation of adipose RAS. Several studies have shown that a fructose diet [23, 53] or a high fat diet [18, 54] in rats could increase the visceral fat pad weight, and subsequent inhibition of RAS activity could reduce visceral fat pad weights and adipocyte sizes. For example, fructose-fed rats exhibited increased adipose expression levels of type 1 Ang II receptors engendered by the upregulation of RAS [53], accompanied by higher weights of epididymal fat pads and adipocyte sizes [23]. Subsequently, RAS blockade by temocapril, an ACE inhibitor, and olmesartan, an ARB, reduced the enlarged adipocyte sizes [23]. These findings suggest that RAS blockade by a renin inhibitor can play a role in regulating visceral adiposity in fructose-fed rats.

## Conclusion

Our data suggest that fructose-fed rats exhibit the significant increases in SBP, serum glucose levels, triglyceride levels and total cholesterol levels, visceral adipose Ang II expression, NOX isoforms expressions, oxidative stress, dysregulated adipocytokines, and visceral adiposity. Treatment with the direct renin inhibitor, aliskiren, significantly reduces the increases in SBP, serum glucose levels, triglyceride levels and total cholesterol levels, dysregulated adipocytokines, and visceral adiposity in high fructose-fed hypertensive rats, and is associated with reducing angiotensin II levels, NOX isoforms expressions and oxidative stress in visceral fat tissues.

## Supporting information

S1 Fig **Effects of aliskiren on (A) food intake and (B) body weight gain in fructose-fed hypertensive rats.** Con: control rats were fed the normal chow diet; Fru: rats were fed the high-fructose diet for 8 weeks; FruA: rats received the same treatment as Group Fru, and aliskiren was concurrently administered; FruB: rats received the same treatment as Group Fru, and aliskiren was administered 4 weeks after the initiation of high-fructose feeding. Values are expressed as means ± SD mean of six independent samples. N = 6 for each group.
(TIF)

S1 Table Angiotensin II levels of the periaortic brown adipose tissue of thoracic aorta and the kidney in the control rats fed with the normal chow diet. (DOC)

## Abbreviations

Ang II: Angiotensin II
ACE inhibitors: Angiotensin-converting enzyme inhibitors
ARB: Ang II type 1 receptor blocker
BW: Body weight
NADPH: Nicotinamide adenine dinucleotide phosphate
NOX: NADPH oxidase
NO: Nitric oxide
PKC: Protein kinase C
RAS: Renin–angiotensin system
SBP: Systolic blood pressure
SOD: Superoxide dismutase
TBARS: Thiobarbituric acid reactive substances
