# Supplementary figures and images for: Renin inhibition improves metabolic syndrome, and reduces angiotensin II levels and oxidative stress in visceral fat tissues in fructose-fed rats
Source: PLoS One. 2017 Jul 10;12(7):e0180712. doi: 10.1371/journal.pone.0180712 (PMC5507254; doi:10.1371/journal.pone.0180712)

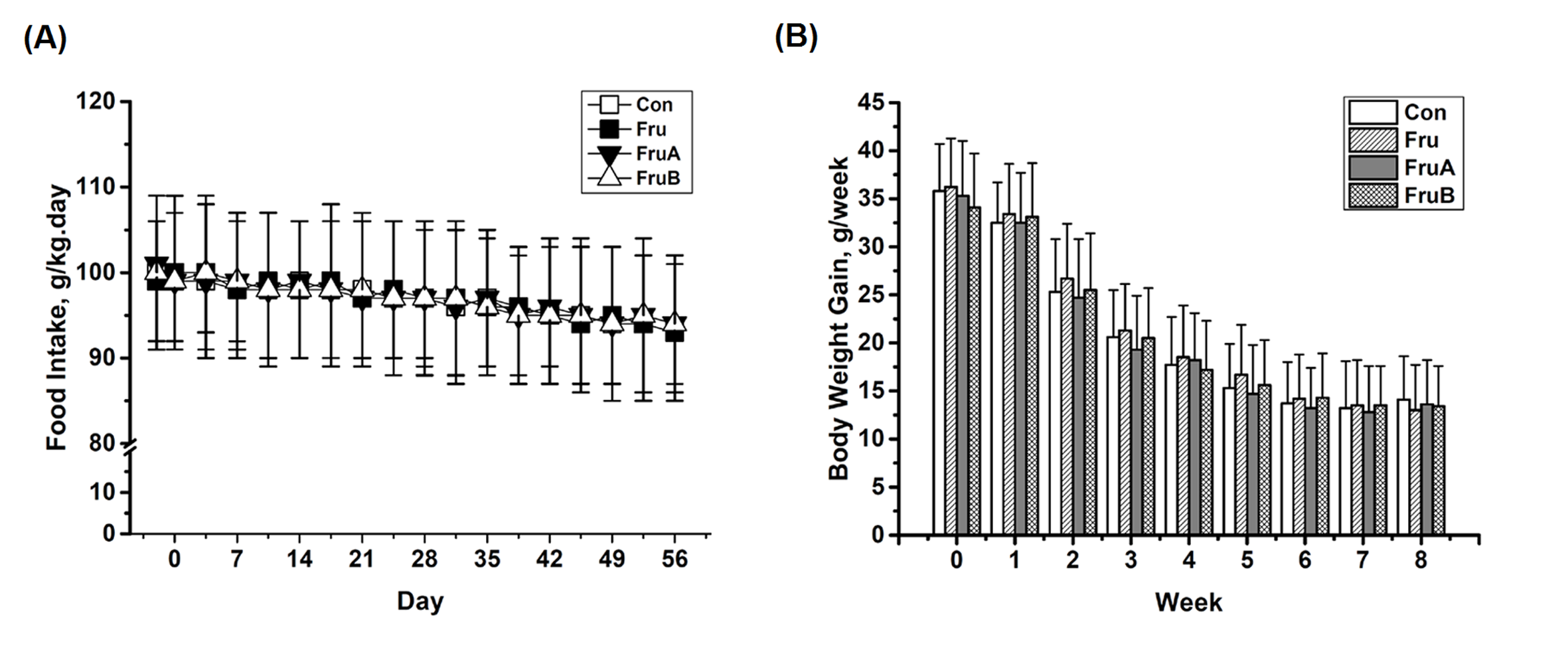

Supplement: S1 Fig — Effects of aliskiren on (A) food intake and (B) body weight gain in fructose-fed hypertensive rats. Con: control rats were fed the normal chow diet; Fru: rats were fed the high-fructose diet for 8 weeks; FruA: rats received the same treatment as Group Fru, and aliskiren was concurrently administered; FruB: rats received the same treatment as Group Fru, and aliskiren was administered 4 weeks after the initiation of high-fructose feeding. Values are expressed as means ± SD mean of six independent samples. N = 6 for each group. (TIF) [file pone.0180712.s001.tif]
