# Supplementary material for: Renin inhibition improves metabolic syndrome, and reduces angiotensin II levels and oxidative stress in visceral fat tissues in fructose-fed rats
Source: PLoS One. 2017 Jul 10;12(7):e0180712. doi: 10.1371/journal.pone.0180712 (PMC5507254; doi:10.1371/journal.pone.0180712)
Supplement: S1 Table — (DOC) [file pone.0180712.s002.doc]

**Supporting Information**

**Supplement** Table 1. Angiotensin II levels of the periaortic brown adipose tissue of thoracic aorta and the kidney in the control rats fed with the normal chow diet.

|  | Periaortic fat from thoracic aorta | Kidney |
| --- | --- | --- |
| Angiotensin II, pg/mg protein | 4.16 ± 0.82 | 62.43 ± 8.75 |

Values are expressed as means ± SD mean of six independent samples. N = 6.
